# Are carnivore digestive separation mechanisms revealed on structure-rich diets?: Faecal inconsistency in dogs (*Canis familiaris*) fed day old chicks

**DOI:** 10.1371/journal.pone.0192741

**Published:** 2018-02-12

**Authors:** Annelies De Cuyper, Marcus Clauss, Myriam Hesta, An Cools, Guido Bosch, Wouter H. Hendriks, Geert P. J. Janssens

**Affiliations:** 1 Laboratory of Animal Nutrition, Department of Nutrition, Genetics and Ethology, Faculty of Veterinary Medicine, Ghent University, Merelbeke, Belgium; 2 Clinic for Zoo Animals, Exotic Pets and Wildlife, University of Zurich, Zurich, Switzerland; 3 Animal Nutrition Group, Department of Animal Sciences, Wageningen University, Wageningen, The Netherlands; 4 Faculty of Veterinary Medicine, Utrecht University, Utrecht, The Netherlands; University of Illinois, UNITED STATES

## Abstract

Pronounced variations in faecal consistency have been described anecdotally for some carnivore species fed a structure-rich diet. Typically two faecal consistencies are distinguished, namely hard and firm versus liquid and viscous faeces. It is possible that a separation mechanism is operating in the carnivore digestive tract, as in many herbivore species. Six beagle dogs were fed two experimental diets in a cross-over design of 7 days. Test diets consisted of chunked day old chicks differing only in particle size (fine = 7.8 mm vs coarse = 13 mm) in order to vary dietary structure. Digestive retention time was measured using titanium oxide (TiO_2_) as marker. The total faecal output was scored for consistency and faecal fermentation profiles were evaluated through faecal short-chain fatty acid (SCFA) and ammonia (NH_3_) analyses. A total of 181 faecal samples were collected. Dietary particle size did not affect faecal consistency, fermentative end products nor mean retention time (MRT). However, a faecal consistency dichotomy was observed with firm faeces (score 2–2.5) and soft faeces (score 4–4.5) being the most frequently occurring consistencies in an almost alternating pattern in every single dog. Firm and soft faeces differed distinctively in fermentative profiles. Although the structure difference between diets did not affect the faecal dichotomy, feeding whole prey provoked the occurrence of the latter which raises suspicion of a digestive separation mechanism in the canine digestive tract. Further faecal characterisation is however required in order to unravel the underlying mechanism.

## Introduction

Separation mechanisms in the digestive tract that selectively retain either fluids or particles have been described in many herbivorous species such as ruminants, lagomorphs, rodents and some birds [1–4]. Typically, the functional existence of these mechanisms is explained either with respect to a comparative delay or acceleration of plant fibre particles to, respectively, enhance their digestion or to rid the digestive tract of them quickly [5] or with respect to a washing of the particulate digesta by fluid in order to direct very fine particles, including microbes, in an aborad or orad direction [6]. In mammalian hindgut fermenters the first principle often occurs when the time-consuming process of fibre fermentation is accounted for by selectively retaining the small, easy-to-ferment plant fibre particles and excreting the larger, coarse, more difficult-to-ferment particles more rapidly from the hindgut [1,7]. Similarly, in some birds, this physical principle occurs with fluids and fine matter being retained in the caeca and coarse, large particles being excreted with the ordinary faeces [1,4,8]. Typically, this results in longer retention times for the fluid fraction and shorter retention times for larger particles [4,9]. In turkey (*Meleagris gallopavo*), this separation in particles leads to the presence of two faecal consistencies—solid vs liquid—with larger particles that tend to be excreted with solid excreta whereas the smaller ones are excreted in more liquid excreta [4] in which the protein level and microbial count is higher [8]. Although not specifically studied to date, there is reason to believe that separation mechanisms are present in carnivores as well. Wolves (*Canis lupus*) fed whole prey produce two types of faeces, i.e. firm, hard faeces and dark, watery, loose faeces, as described by Floyd et al. (1987) [10], Weaver (1993) [11], Ruehe et al. (2003) [12] and Jethva and Jhala (2004) [13]. The liquid faeces are considered non-collectable and therefore are not included in faecal analyses to evaluate the feeding ecology of wild wolves. Similarly, a discrepancy in faecal consistency has been observed when feeding cheetahs (*Acinonyx jubatus*) whole prey, with collectable faeces described as hard to soft and non-collectable faeces as viscous [14,15]. To our knowledge, the systematic occurrence of two faecal consistencies within a diet has not been reported in scientific literature in healthy domestic carnivores fed commercially prepared diets (from dry kibble diets to processed meat). Only Hill et al. (2011) [16] observed that the water content of faeces and looser (watery) faeces, was higher in the afternoon than in the morning in dogs fed canned diets containing texturised vegetable protein from soya in morning meals, which was attributed to the soy carbohydrates present in the texturised vegetable protein. Based on these reports, we speculate that the occurrence of two types of faeces might be an indication of a separation mechanism operating in the gastrointestinal tract which might be linked to different substances in a heterogeneous carnivore diet. Examples of more recalcitrant substances are skin, hair, bone or collagen in whole prey (i.e., 'animal fibre' [17]), which may have some analogies with the coarse or larger-sized, difficult-to-digest plant material consumed by herbivorous species. As in plant-derived fibre, more soluble and insoluble fractions can be distinguished within 'animal fibre', with collagen representing the soluble, smaller particles and fermentable fraction and substances such as hairs and bones as the more insoluble, coarser fraction [18], which could provoke a possible separation in the gut as described above for the herbivorous species. Therefore, as a first step, we wanted to evaluate how the digestive physiology of the dog, as a carnivore species, is affected when fed a whole prey diet. As particle size may impact the separation efficiencies [2] we included this as a dietary contrast in our study design. Insight in the digestive physiology was obtained by monitoring faecal patterns and associations between faecal consistency with retention time and faecal fermentation profiles.

## Material and methods

### Experimental design and diet (*based on De Cuyper et al. (2017)* [19])

Experimental procedures were approved by the Ethical Committee of the Faculty of Veterinary Medicine of Ghent University (EC2015/45). Dogs were housed in individual adjacent kennels consisting of an indoor (90 cm x 473 cm) and outdoor part (90 cm x 300 cm). Kennels were enriched with toys and dogs were looked after daily by animal caretakers and trial leader with short free roaming moments in the dog facility. Six adult beagle dogs (four females and two males) with an average (± standard deviation (s.d.)) body weight of 10.1 kg (± 1.1), a body condition score between 3 and 5 on a scale of 1 (anorexic) to 9 (obese), and aged between 2 and 7 y, were fed two test diets in a cross-over design of 7 d per period. Both test diets were based on exclusively day-old chicks (Kiezebrink Putten B.V., Hoge Eng Oost, the Netherlands) minced at a die size of 7.8 mm for the fine diet or 13 mm for the coarse diet (KOLBE AW 130 meat mincer). This was the largest contrast that could be obtained within the limitations of the available food processing equipment. It was assumed that this contrast in die size would create a sufficiently large contrast in particle size. Because of the limited duration of the trial, the diets were not adjusted for any potential deviations from nutrient guidelines, in order to keep the intervention simple.

In order to adapt the dogs to the chunked day-old chicks, a 3-wk dietary adaptation period was provided before the actual start of the trial. In the first week, chunked day-old chicks (13 mm) were gradually added to the routinely fed kibble diet (fulfilling maintenance energy requirements (MER) for adult laboratory dogs [20]).

In the consecutive two weeks, chunked day-old chicks were meal-fed (100% MER) to maintain constant body weight. Only one dog was often reluctant to eat its whole meal whereupon refusals were offered again at a later time point of the day. After the adaptation period, the cross-over trial was executed with dogs being meal-fed once between 8 AM and 9 AM every day with each dog always receiving the same amount of food throughout the cross-over experiment hence avoiding differences in food intake between dietary treatments. All dogs had *ad libitum* water access and were weighed weekly. A total faecal collection was carried out for every dog during the cross-over trial (6 days for period 1 and 7 days for period 2; the difference in period length was taken into account for frequency calculations, see below). Each kennel was checked every 15 min day and night for defecation events and the time of each defecation was recorded.

### Patterns of faecal consistency

Before collection, the faecal consistency was scored for every sample using the Waltham faeces scoring system [21] based on visual appearance. The scoring scale runs from 1 to 5 with 1 being 'hard, dry and crumbly faeces' and 5 being 'watery diarrhea'. Half-scores were used, giving a total of 9 possible categories. Faecal samples were weighed, frozen at –20°C and dried afterwards at 60°C to constant weight for determination of the dry matter (DM) content.

### Transit time

Mean retention time (MRT) and maximum retention time (MaxRT) were determined for each treatment by adding 2 g TiO_2_ (VWR, International BVBA, Leuven, Belgium) per kg of diet on the fifth day of every test period. The marker was poured upon the diet per dog and was mixed manually and thoroughly with the diet to ensure homogenous distribution of the marker. Faecal samples collected from one day before TiO_2_ addition until two days after the TiO_2_ addition were used for Ti analysis. All samples were scored (see above), weighed and dried at 60°C.

### Fermentation products

In order to analyse the microbial fermentation products, fresh faecal subsamples (n = 61) were collected within 15 min of defecation for every dog on the third and fourth day of every test period. After scoring the faecal consistency (see above), pH was measured with a calibrated portable pH meter (HI 99141, pH electrode probe HI 72911, Hannah Instruments, Belgium). Afterwards, a representative aliquot of faeces was collected from every sample for short-chain fatty acid (SCFA; including branched-chain fatty acids (BCFA)) and NH_3_ analyses. All fresh faecal samples were stored at -20°C until further analyses.

### Chemical analyses

Dietary DM was determined by drying to constant weight at 103°C. Ash content was determined by combustion at 550°C. Crude protein (6.25 × N) was analysed using the Kjeldahl method [22] and crude fat was analysed according to the Soxhlet method (with and without pre-hydrolysis of samples) [23]. Crude fibre was analysed by acid-alkali digestion [24]. Total fibrous matter and insoluble fibre were analysed according to the method of Cools et al. (2015) [25]. This method is based on the *in vitro* digestive simulation of Boisen and Fernández (1995) and Hervera et al. (2007) [26,27] and resembles the total dietary fibre (TDF) analysis according to Prosky et al. (1985) [28] with the difference that the fibre fraction obtained includes not only the plant-derived carbohydrate fraction (TDF) but also animal fibre (protein-rich). Titanium in faeces was analysed according to the method of Myers et al. (2004) [29]. For determination of SCFA and NH_3_, ca. 0.5–1.0 g faeces was added to safe-lock tubes (2 ml; Eppendorf AG, Hamburg, Germany) containing 1 ml of a 0.0333 M H_3_PO_4_ solution (for SCFA) or 1 ml of 10% TCA solution (for NH_3_). The content of the tubes was mixed on a vortex for ca. 3 sec and weighed. The mixed samples were centrifuged at 15,000 rpm for 5 min at 4°C (Centrifuge 5417R, Eppendorf AG). The sample supernatant was analysed for SCFA (acetic, propionic, isobutyric, butyric, isovaleric and valeric acids) and NH_3_ concentrations following Bosch et al. (2008) [30].

### Calculations

The MRT of TiO_2_, the best single measure of rate of passage through the gastrointestinal tract, was calculated according to Thielemans et al. (1978) [31].

MRT(h)=ΣtiCiΔti/ΣCiΔti

where C*i* is the marker concentration in the interval indicated by time t*i* (hours after marker administration) and Δt*i* = the interval of the concerning sample:

Δti=((ti+1−ti)+(ti−ti−1))/2

Furthermore, the time of last marker excretion (MaxRT) (< 5% of the peak concentration) was determined for both treatments. Additionally, marker excreta concentrations were plotted over time with concentrations expressed as the percent of the marker peak concentration [32].

In order to explore any difference in marker excretion between 'firm' (score 1 to 3.5) and 'soft' faeces (score 4 to 5) (see above), the percent of the marker peak concentration was labelled firm or soft.

Frequencies of every single faecal score were calculated per diet. Second, the average number of defecations per day and the average faecal score per day were calculated per dog and per diet. Faecal scores were plotted over time per dog for the whole trial in order to explore faecal consistency data. Furthermore, faecal score frequencies were visualized using histograms for both dietary treatments. Additionally, a subdivision in faecal scores was made to firm and soft as indicated above. The number of firm and soft faeces per day and the ratio soft to firm faeces were calculated per dog and per diet. The SCFA and NH_3_ were expressed on a DM basis. Furthermore, BCFA (isobutyric and isovaleric acid) was expressed as the percentage of the total SCFA [33].

### Statistical analyses

The effect of dietary treatment on faecal SCFA, NH_3_, and DM concentrations and pH values was evaluated using a linear mixed effect model (lmer function of the lme4 package in RStudio) with dietary treatment, period and group (order of dietary treatments) as fixed effects and dog as a random effect. Additionally, the faecal score was included as a continuous fixed effect in the model. The interaction between faecal score and dietary treatment was also included in the model, except when P > 0.10 (relationships were considered trends when 0.05 < P < 0.10), then the interaction was omitted from the model. Results are reported as regression estimates.

Pearson correlations were determined for the following relationships: DM concentrations versus faecal score; average faecal score per day versus average number of defecations per day; the number of soft faeces per day, the number of firm faeces per day and the ratio soft faeces:firm faeces versus MRT and also versus MaxRT. Relationships were considered trends when 0.05 < P < 0.10.

## Results

All dogs remained healthy throughout the study. A general decrease in bodyweight was observed for all dogs throughout the cross-over trial (approximately 3% bodyweight loss). All provided food was consumed every day. Only one dog showed reluctance to eat its whole meal at once. Refusals were offered again at a later time point during the day except during retention time testing on the fifth day of the first test period. Subsequently, on the fifth day of the second test period, this dog was offered the same diminished amount of food in order to compare test periods (356 g instead of 808 g). The chunked day old chicks contained 38% amount of total fibrous matter and 26.2 insoluble fibrous matter (on a DM basis) (Table 1).

**Table 1:** Analysed components and calculated energy content of chunked day old chicks.

**Component (% of DM)**^a^	
Dry matter (% as is)	24.9
Crude protein	57.3
Crude fat	22.7–26.4^b^
Total fibrous matter	38.0
Insoluble fibre	26.2
Crude ash	7.1
Crude fibre	2.5
**Metabolisable energy (kJ/100 g DM)**^c^	1672

DM = dry matter

^a^ Unless otherwise stated

^b^ Smallest value without hydrolysis, largest value with hydrolysis

^c^ The metabolisable energy is the average of the values calculated by Atwater factors (16.7 × crude protein + 37.7 × crude fat + 16.7 × NfE) and the alternative predictive equation of the NRC (2006) with NfE (Nitrogen free extract) calculated as 100 - moisture% - crude protein% - crude fat% - crude fibre% - crude ash%

### Patterns of faecal consistency

**Fig 1:**
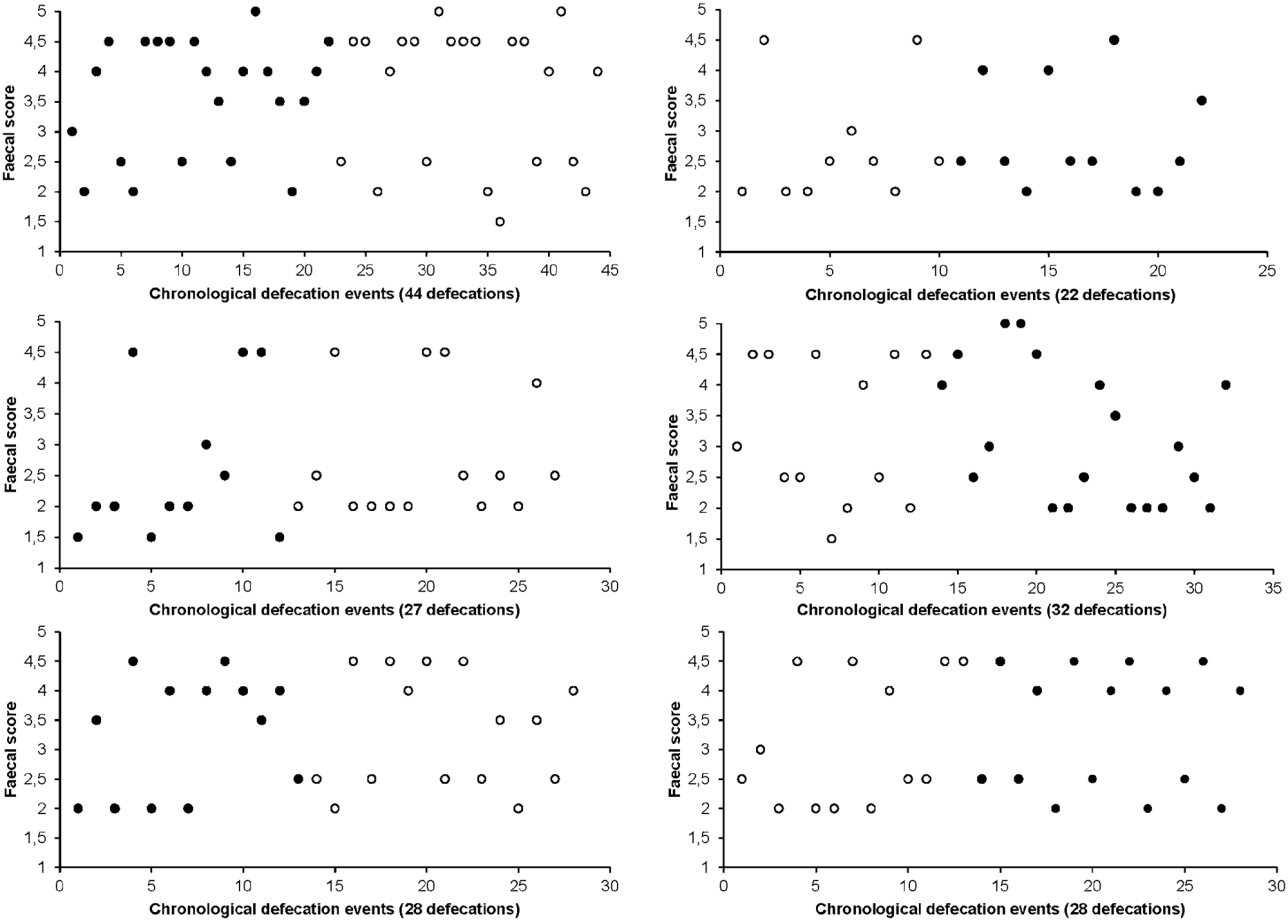
Chronological pattern of faecal consistency scores of six beagle dogs during the cross-over trial. Black circles = fine diet; white circles = coarse diet. The faecal collection was carried out for 6 days in period 1 and 7 days in period 2.

**Fig 2:**
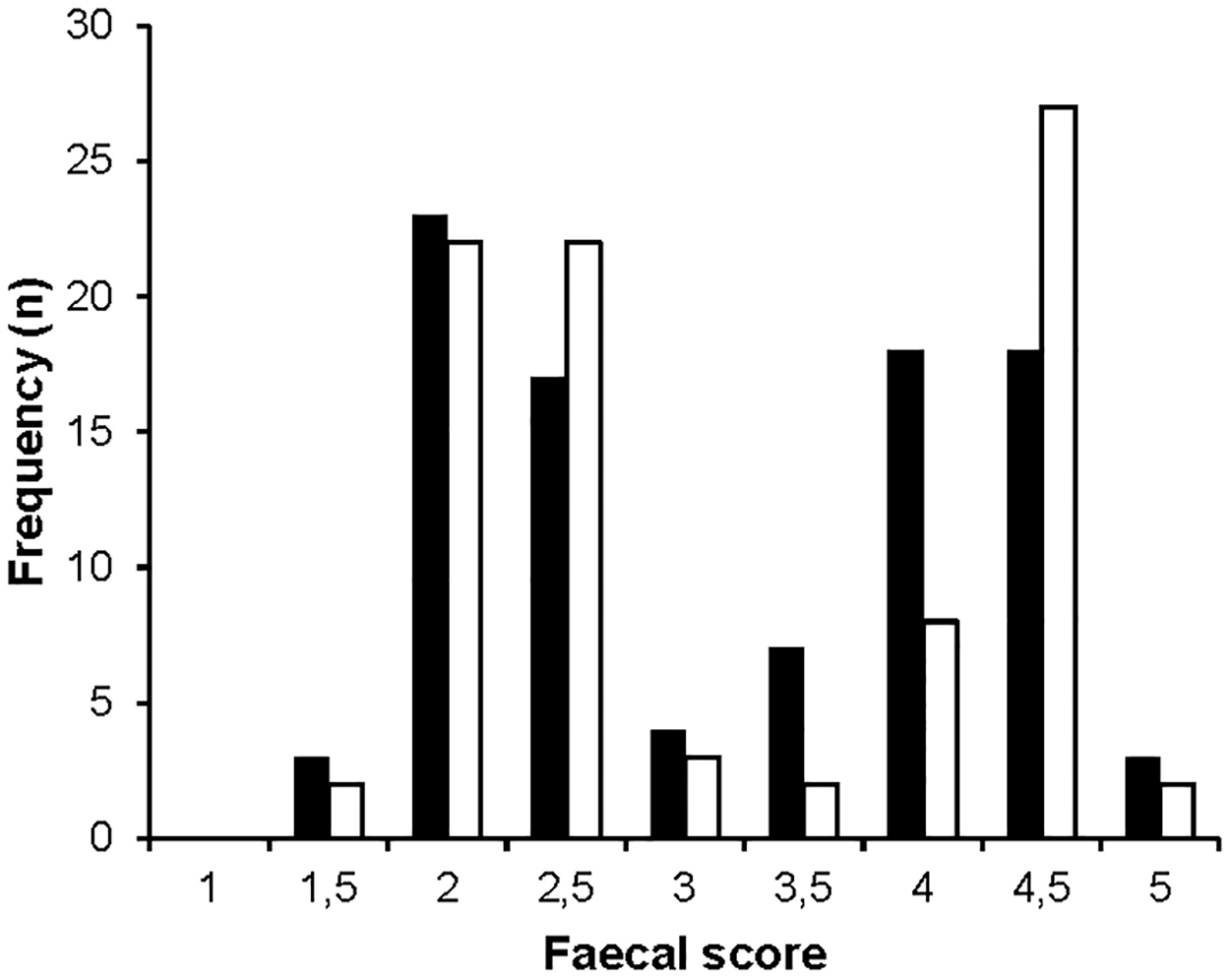
Frequency distribution of all faecal scores per dietary treatment. Black bars = fine diet (n = 93 defecations); white bars = coarse diet (n = 88 defecations).

A total of 181 faecal samples were collected. Liquid faeces (≥ score 4) were collected as completely as possible. The DM content negatively correlated with faecal score (R = -0.719, P < 0.001). By observing faecal score patterns over time for every dog, a dichotomy of firm and soft faeces within individuals became obvious, independently of the time of day. Fig 1 shows individual faecal patterns of two dogs included in the experiment. When faecal scores were expressed as a frequency per diet (Fig 2), the same pattern occurred with the scores 2–2.5 and 4–4.5 being the most frequently observed scores. The average number of soft faeces per day, firm faeces per day, the ratio soft faeces to firm faeces can be found in Table 2.

**Table 2:** Average daily number of defecations, average daily faecal score, frequencies of faecal consistencies and transit times for 6 beagle dogs fed a fine or coarse diets in a cross-over design.

Parameter	Fine diet		Coarse diet	
	Mean	s.d.	Mean	s.d.
**Defecations/d**	2.4	0.70	2.3	0.42
**Faecal score/d**	3	0.34	3	0.34
**Soft faeces** (n/d)	1.0	0.56	0.93	0.57
**Firm faeces** (n/d)	1.4	0.29	1.3	0.15
**Ratio soft/firm**	0.74	0.39	0.75	0.54
**MRT** (hrs)	19.5	5.0	22.0	3.8
**MaxRT** (hrs)	30.8	10.6	33.3	9.6

s.d. = standard deviation; n = number; MRT = mean retention time; MaxRT = maximum retention time

### Transit time

**Fig 3:**
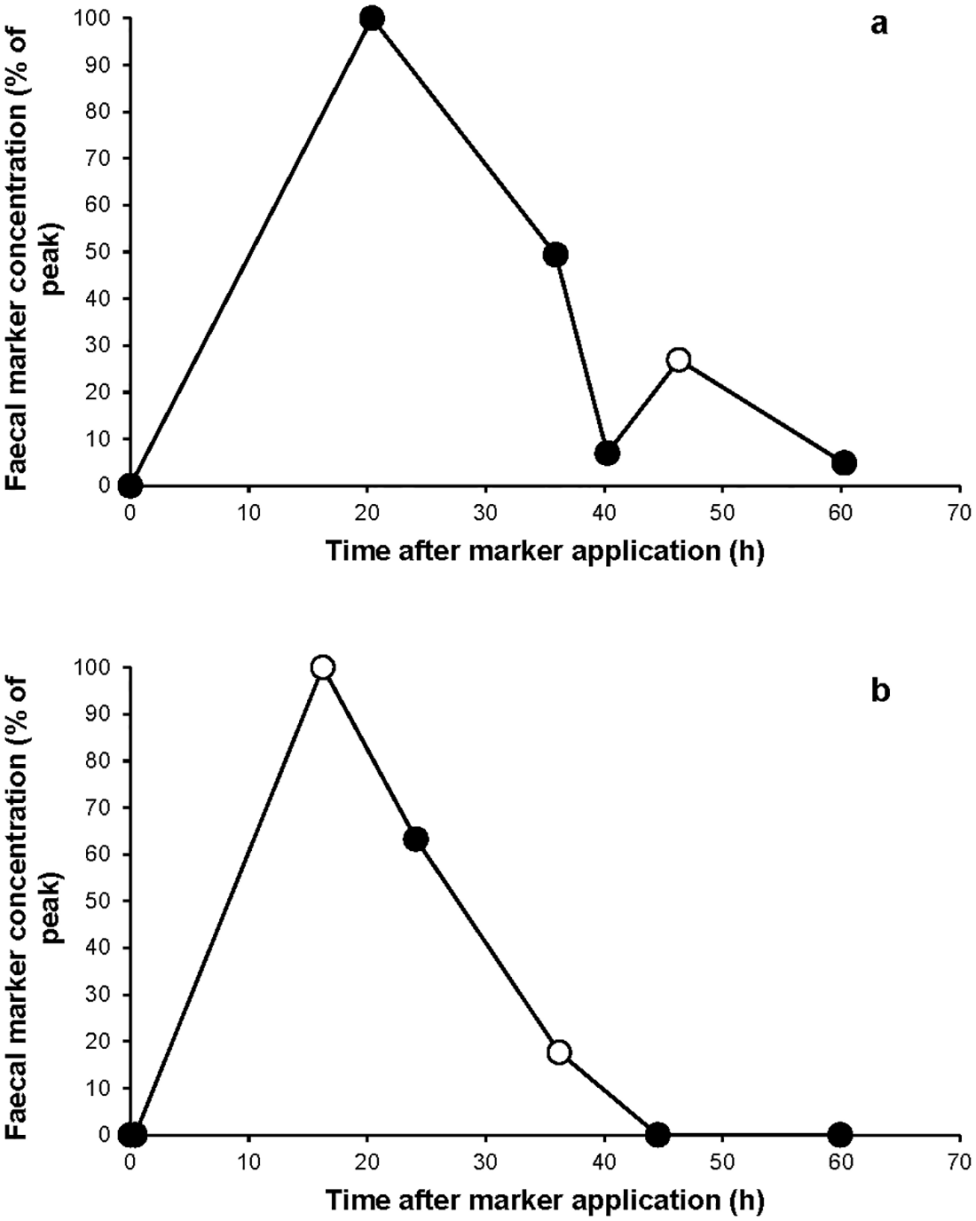
Exemplary marker excretion patterns of the TiO_2_ marker for two beagle dogs (Marker excretion patterns of all 6 beagle dogs can be found in S1 Fig). Black circles = firm faeces (faecal score 1 to 3.5); White circles = soft faeces (faecal score 4 to 5); Graph a showing marker excretion pattern for one beagle dog on the coarse diet with two separate marker peaks; Graph b showing marker excretion pattern for one beagle dog on the coarse diet with one single marker peak.

The TiO_2_ recovery averaged at 81.2% (s.d. = 12.9) for the fine diet and 73.7% (s.d. = 8.2) for the coarse diet. The average MRT and MaxRT values are presented per diet in Table 2. Marker excretion patterns showed a single peak followed by a continuous decline without a difference between firm and soft faeces for all dogs on both diets, except for one dog that showed a recurrent peak of marker for soft faeces (Fig 3a).

### Fermentation products

Dietary treatment, period and group had no effect on DM, NH_3_ and SCFA concentrations, except for butyric acid which was affected by dietary treatment (P = 0.04) and for which a tendency towards an interaction between treatment and faecal score was observed (P = 0.06). The pH values tended to be affected by dietary treatment (P = 0.05). Faecal score was not found to relate to variation in butyric acid and isovaleric acid. As faecal score increased, NH_3_ (P = 0.02), acetic acid (P < 0.001) and valeric acid concentrations (P < 0.001) increased, whereas propionic acid (P = 0.02) and isobutyric (P = 0.001) concentrations decreased. Faecal pH decreased with faecal score (P < 0.001) (Table 3).

**Table 3:** Regression estimates (± s.e.) for faecal DM, short chain fatty acid (SCFA) and ammonia (NH_3_) concentrations and faecal pH values from 6 beagle dogs fed a fine or coarse diet in a latin square cross-over design. In the linear mixed effect model the fine diet was considered as the reference for Treatment and the diet order fine followed by coarse as the reference for Group.

Parameter	Intercept	Treatment	Period	Group	Faecal score
**DM** (g/kg)	530.6***	-4.9	-2.8	-19.3	-54.0***
	(± 43.8)	(± 11.3)	(± 11.3)	(± 19.9)	(± 5.4)
**SCFA** (mmol/kg DM)					
**Acetic acid**	39.6	-0.61	3.5	-9.9	19.0***
	(± 24.2)	(± 6.3)	(± 6.3)	(± 10.9)	(± 3.0)
**Propionic acid**	62.3**	-3.7	4.5	-2.0	-5.0*
	(± 22.0)	(± 4.6)	(± 4.6)	(± 11.5)	(± 2.2)
**Butyric acid**	49.4**	-16.0*	-1.0	-1.2	-5.1
	(± 16.4)	(± 7.9)	(± 2.4)	(± 4.9)	(± 3.7)
**Valeric acid**	0.90	0.14	0.09	0.20	0.64***
	(± 0.62)	(± 0.20)	(± 0.20)	(± 0.20)	(± 0.09)
**Isobutyric acid**	7.2*	-0.49	1.4	1.3	-1.2**
	(± 3.7)	(± 0.75)	(± 0.75)	(± 1.9)	(± 0.36)
**Isovaleric acid**	8.6*	-0.38	1.3	0.65	-0.23
	(± 3.4)	(± 0.80)	(± 0.80)	(± 1.7)	(± 0.38)
**NH**_**3**_ (g/kg DM)	2.0	-0.21	0.03	-0.29	0.30*
	(± 1.0)	(± 0.26)	(± 0.26)	(± 0.47)	(± 0.12)
**pH**	7.4***	0.18	0.12	0.13	-0.22*
	(± 0.43)	(± 0.09)	(± 0.09)	(± 0.22)	(± 0.04)

* = P < 0.05;

** = P < 0.01;

*** = P < 0.001;

Relationships were considered trends when 0.05 < P < 0.10.

### Correlations

**Fig 4:**
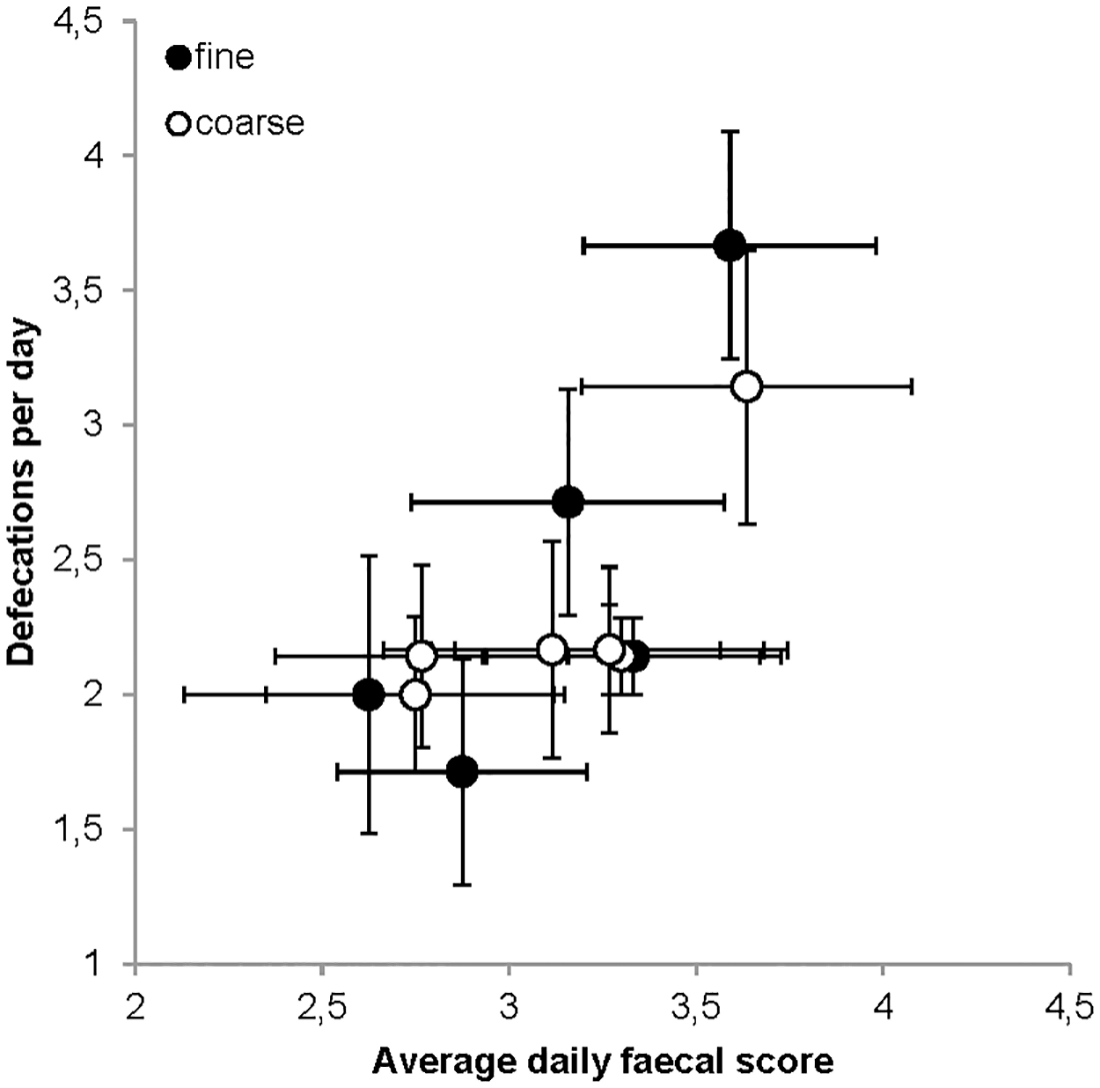
Average number of defecations per day vs the daily faecal score for both dietary treatments. Black circles = fine diet; white circles = coarse diet; n = 6.

**Fig 5:**
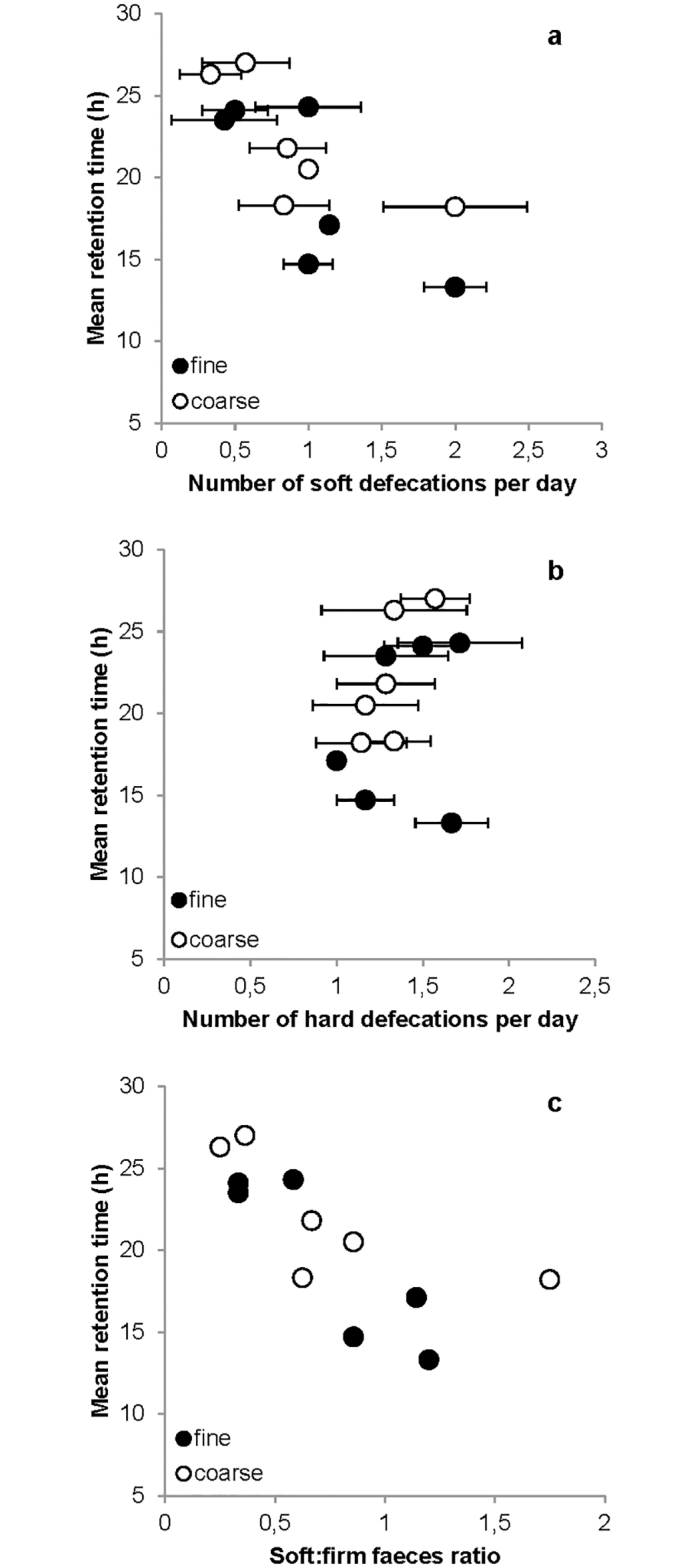
Mean retention time vs the average daily number of soft and firm faeces and soft:firm ratio produced for both dietary treatments. Black circles = fine diet; white circles = coarse diet; n = 6.

Across dogs, the average number of defecations per day tended towards a positive correlation with the average daily faecal score for the fine diet (R = 0.733; P = 0.097) and the coarse diet (R = 0.774; P = 0.071) (Fig 4; Table 2), i.e. dogs with a higher frequency of soft faeces had a larger number of defecations. The number of soft faeces produced per day tended to be negatively correlated with the MRT for the fine diet (R = -0.780; P = 0.067) as well as the coarse diet (R = -0.739; P = 0.093), i.e. dogs with a higher frequency of soft faeces had shorter retention times (Fig 5a). Similarly, the number of soft faeces produced per day was negatively correlated to the MaxRT for the fine (R = -0.898; P = 0.015) and the coarse diet (R = -0.886; P = 0.019). The soft:firm faeces was negatively correlated to the MRT for the fine diet (R = -0.887; P = 0.018) but only tended towards a negative correlation on the coarse diet (R = -0.735; P = 0.096) (Fig 5c). Correlations between the soft:firm faeces and MaxRT tended to be negative for the fine diet (R = -0.807; P = 0.052) and were negatively correlated for the coarse diet (R = -0.853; P = 0.031). No significant correlations were found between the number of firm faeces per day and MRT (Fig 5b) or MaxRT.

## Discussion

### Faecal consistency

This study provides a first insight in the occurrence of a faecal consistency dichotomy in canines fed a structure-rich whole prey-like diet. Dogs fed whole prey diets (day old chicks) produced two types of faeces in terms of consistency with concomitant differences in DM concentration: soft, more liquid faeces (with a score around 4–4.5) alternated with firm, hard faeces (with a score around 2–2.5). Although this was not different between the two dietary treatments, i.e. a slight structure difference (fine vs coarse chicks) did not affect this observation, this was in contrast with the normal defecation pattern preceeding the experiment when dogs were fed a commercial dry kibble diet (Hill's Science Plan Advanced Fitness, 1570 kJ/100 g) and had more consistent faecal consistencies (ADC, personal observation). The absence of a dietary effect (fine vs coarse) probably lies in the particle size difference, which was only 5.2 mm and probably too small to have a relevant effect. However, when considering studies in herbivores and birds, particle size differences of μm's or mm's have been shown to affect gut retention times [34,35]. Given the absence of a frame of reference concerning dietary particle size in carnivores, a particle size difference of a mm difference range was hypothesized to provoke an effect. One could state that particle size might have been undone due to chewing on the food. However, the dogs in this study tended to gorge feed, as does their wild ancestor the wolf [36], on the chick diet which makes this statement unlikely. The occurrence of a faecal dichotomy in dogs when fed day old chicks compared to the absence of a consistency duality when fed their traditional pelleted diet, makes it likely that structure does provoke the faecal dichotomy. Day old chicks are naturally rich in animal fibre containing soluble and recalcitrant insoluble coarse substances which we postulate are key in eliciting the faecal dichotomy. The findings from Hill et al. (2011) [16], i.e. different moisture contents in faecal droppings from dogs fed a diet enriched with texturised vegetable protein (TVP), do not seem to abide with the 'structure' hypothesis. However, TVP typically consist of protein but also 30% of indigestible carbohydrates that may serve as fermentation substrates in the hindgut and that are suggested to cause faecal moisture differences (see below). Given the fact that a consistency difference seemingly does not occur in less structurized diets, and that firm and soft faeces seemed to differ in the amount of animal fibre present (feathers, visual observation), a faecal dichotomy seems to associate with structure.

Faecal consistency dichotomies have been described for wild carnivores in captivity. Wolves (*Canis lupus*) and cheetahs (*Acinonyx jubatus*) both have been described as defecating 'collectable' (= firm, hard) and 'non-collectable' (soft, liquid or viscous) faeces when fed whole prey [10–15]. When feeding whole prey to leopards (*P*. *pardus*) [37], liquid faeces were sometimes produced next to the commonly collected firm faeces (Lumetsberger T., personal communication). Additionally, it has been shown that the water content of faeces is higher in the afternoon than in the morning in morning-fed dogs consuming canned diets containing texturised vegetable protein from soya [16]. To our knowledge, the intra-individual dichotomy of two types of faecal consistencies on a carnivorous diet has not been reported in any other studies than those for the wolf, cheetah, leopard and dog. A large number of studies investigated the effect of different diets on, amongst other factors, faecal consistency in various domestic and wild carnivores including the bobcat (*Felis rufus*), cheetah (*Acinonyx jubatus*), tiger (*Panthera tigris*), jaguar (*Panthera onca*), African wildcat (*Felis lybica*), domestic cat (*Felis catus*) and domestic dog (*Canis familiaris*) (e.g. [38–45]). However, authors did not specifically report on profound intra-individual differences in faecal consistency when a carnivore was fed a specific study diet. It is possible that the intra-individual dichotomies in faecal consistency was not elicited by the specific diets in these studies, it was left unnoticed or it is not a common feature in carnivore digestive physiology. Focussing on dogs, several studies (e.g. [46–49]) investigated the effect of several dietary compositions on faecal consistency (e.g. firmer faeces in German Shorthair Pointers fed with a chicken canned diet compared to dry chicken diet and dry and canned beef diets [46]. However no intra-individual alternating pattern of two faecal consistencies are reported in those studies. Careful recording of its absence as well as presence in future studies in other carnivorous species will allow further exploration of the variation in this aspect of digestive physiology.

### How do soft faeces come about?

Excluding infectious diarrhea, the occurence of soft faeces or loose stools in dogs as such, has been subject to some debate. Rolfe et al. (2002) [50] mentioned that with a shorter transit time, the capacity to absorb water and electrolytes in the colon becomes impeded and leads to the production of softer, loose stools (with moisture and consistency being closely related [51,52]). However, others state that water and electrolyte absorption are not the strongest determinant for faecal moisture, instead higher fermentation activities due to a longer residence time in the colon leading to an osmotic imbalance can be responsible for a higher faecal score [53–56]. The tendency towards a negative correlation between the daily number of soft faeces and the MRT and the negative correlation between the daily number of soft faeces and the MaxRT implies that at shorter overall retention times, more soft faeces were defecated (Fig 5). Our individual dogs hence might have differed in the extent to which softer digesta components were either directly defecated, or retained in the colon for water re-absorption. However, given the fact that the insoluble powder marker used (TiO2) associates with the solid fraction of the diets, it seems dubious to relate retention times obtained with solid markers to the frequency of soft, liquid stools. One specific and peculiar observation in dogs that should be adressed is the lower faecal quality, in other words looser stools in large and giant dog breeds (e.g. great Dane) compared to smaller ones [55]. The authors suggested that the latter occurred due to a longer colonic residence time in larger dogs which allows for more fermentation, hence, more 'osmotic pressure' attracting more water [56]. In another study, the authors suggested that the higher faecal moisture in large breeds might have to do with a higher permeability in the small intestine of large breed dogs [57].

### Possible underlying mechanisms of the faecal dichotomy

Apart from questioning how soft stools come about, which has been subject of many studies (see above), the occurrence of a faecal consistency dichotomy within dogs suggests additional mechanisms operating in the gut. Differences in fermentation profiles between the observed faecal consistencies were present, which suggests gastrointestinal separation of substances with distinct fermentation properties. As faeces were softer, NH_3_, acetic acid and valeric acid concentrations were higher whereas propionic acid and isobutyric acid concentrations as well as pH values were lower compared to firmer faeces. The fibre type present in the experimental diets was exclusively animal fibre and thus protein-rich (total fibrous matter = 38.0% of DM; insoluble fibrous matter = 26.2% of DM). Faecal SCFA and ammonia concentrations were comparable to the levels found in domestic dogs fed commercial diets rich in plant-derived fibre [58,59]. This suggests that the undigested parts of the chick diet can serve as a source for SCFA production as shown in humans and cheetahs [17,18,60] with different animal based substrates that have different fermentative profiles [17,18]. Based on the ratios acetic acid, propionic acid and butyric acid to total SCFA from our study and the ratios from in vitro fermentation of animal-based substrates [18], collagen, cartilage and glucosamine-chondroitine were potentially substrates for fermentation in the undigested parts of the chick. The higher acetic acid concentration in the soft faeces type suggests more fermentation in the soft than the firm faeces type. It would typically be attributed to carbohydrate fermentation, but can also be generated by protein fermentation [60,61]. Ammonia and valeric acid concentrations, which are protein fermentation indicators [60,61], were higher for soft stools, suggesting a higher level of protein fermentation in softer faeces. However, such proteolytic fermentation is also associated with increased propionic acid and BCFA concentrations (isovaleric and isobutyric acid) [60], which was not found in the present study and therefore do not support that acetic acid concentration was higher because of protein fermentation. Faecal pH decreased with faecal score, which is typically to be expected when SCFA and the the alpha-hydroxy acid lactate are produced [62,63]. Yet, the only measured SCFA that increased in the soft faeces type was acetic acid, a weak acid [63]. Therefore, we suspect that the lower pH in the soft faeces type is caused by the production of lactate, a stronger acid than the SCFA. Lactate can cause a significant decrease in pH which can inhibit production of SCFA, except for acetic acid that can be, under certain circumstances, high [62]. Although animal fibre typically consists of indigestible proteins, substances such as chondroitin sulphate contain glucose chains [64] and may serve as substrates for lactate production [65]. In many digestive systems, lactate is easily converted to propionate, with a prominent role for Bacteroidetes (termites [66]; humans [67]). However, in cheetahs fed a whole prey diet, strains of Bacteroidetes were very low in numbers [68], which allows speculation that lactate concentrations might have been high in the caecum and lowered the pH in the absence of conversion to propionate. We therefore recommend to measure faecal lactate concentrations in softer faeces in future studies.

Several mechanisms might explain the observation of different faecal consistencies. One could bluntly state that the softer stools are just caused by infectious diarrhea. Raw meat diets can be associated with infectious agents and can impair the health of the animal [69]. The day old chicks used in this dissertation were evaluated for pathogenic bacteria and the amount of Enterobacteriaceae was relatively high. However, day old chicks tested negative for *Salmonella* spp. and dogs remained clinically healthy throughout the study. Additionally, this diarrhea would make it impossible for dogs to produce alternating firm faeces which are not indicative for diarrhea, hence suggesting that observations are of a physiological rather than pathological kind.

The liquid, runny faeces observed in wolves when fed whole prey [10–13, 70] have previously been associated with the ingestion of large protein-rich meals (feast meals). The digesta would pass quickly through the gastrointestinal tract, possibly leading to osmotic imbalance, stimulation of secretion and gut motility, and inhibition of nitrogen and water absorption, which would all lead to increased water content in the faeces [70]. This could be a plausible explanation; the overload of (digestible and undigestible) protein ingestion might end up in the hindgut and cause excess protein fermentation which in its turn may cause watery faeces (osmotic imbalance) [56,71]. The first watery faeces are said to reflect the first meal of the wolves, being a large amount of muscles and organs, hence resulting in runny faeces. Afterwards, when wolves switch to the more indigestible parts of a carcass (i.e. fur, hairs), the faeces are of a firmer consistency [70]. However, one important factor to consider here is that the faecal consistency dichotomy is already caused at the level of prey intake, i.e. selection and ingestion of different prey parts over time. This is in contrast with the methodology used in the present study, where dogs were fed chunked day old chicks, which caused an equal spread of prey parts in one meal. As such, the dichotomy observed is caused within the animal rather than at the level of diet selection, and hence a separation mechanism in the gut is required.

It might be that the stomach plays a regulating role. It is known for dogs that objects of different size differ in the time at which they leave the stomach. Once exceeding a threshold of ca. 5 mm diameter, non-food particles are retained in the stomach until the interdigestive migratory myoelectric complex (IMMC) occurs, which drives large particles towards the duodenum [72,73]. This could lead to a separation of different sized digesta particles over time. As such it seems plausible to think that the substances such as feathers and bones of the chick diet stayed behind in the stomach and were released later on during digestion. However, studying passage of whole prey in the serval (*Felis serval*) and black backed jackal (*Canis mesomelas*), it seemed that substances such as teeth and bones were released with the first defecations [74]. This would be in contrast with the assumption of a retention of large indigestible prey parts in the stomach. Additionally, others could not prove that faecal moisture and consistency were linked to upper gastrointestinal transit (e.g. gastric emptying time) [75]. In this experiment, gastric emptying time and other transit parameters were measured using two marker systems (reported in De Cuyper et al. (2017) [19]). Gastric emptying (13.7 h on coarse diet; 15.4 h on fine diet) was not affected by dietary particle size probably because of the small particle difference between diets and the fact that both diets will have acted as a coarsely chunked diet. Additionally, the marker system used (a wireless motility capsule) will have acted as a coarse particle which might have lacked precision to study the fine diet. In our study, one male (group 1) and one female (group 2) were still intact, hence it could be that sexual hormones influenced gastrointestinal transit parameters. However, in literature, findings on the effect of sex or sexual related hormones on gastric emptying and total transit are contradictory in humans [76–79] and have been reported as absent in dogs [80] and cats [81].

Separation mechanisms in the hindgut are common physiology in herbivores (lagomorphs, rodents and horses) [1,82] and some birds [3,4]. A typical strategy used in hindgut fermenters to account for the time-consuming process of plant particle fermentation is to selectively retain the small, easy-to-digest particles and to excrete the larger, bulky, more difficult-to-digest particles more rapidly from the hindgut [1,7]. In some birds, fluids and small particles can be retained in the caeca and larger particles are excreted with ordinary droppings [1,4,82]. In turkey (*Meleagris gallopavo*), this mechanism has been associated with the occurrence of two faecal consistencies: solid faeces including large particles and liquid faeces including small particles [4]. Given the analogies of plant fibre and animal fibre [17,18], i.e. recalcitrant substances such as hair, bone, feathers might compare to insoluble, coarse plant fibres (e.g. cellulose), it could be beneficial to accelerate the excretion of coarse, indigestible animal fibres from the carnivore gut. This would imply that easy-fermentable and soluble animal fibres (collagen) would reside longer in the colon. The fermentative profiles for firm and soft faeces were clearly distinct with higher indicators for protein fermentation in soft stools (i.e. higher concentrations of SCFA and NH3). Long retention in the colon of digesta can lead to high fermentation activities which in turn might lead to higher faecal scores due to an osmotic imbalance (see above). As such, the latter seems explenatory for consistency observations: soft stools with high amounts of fermentation indicators were retained longer in the colon, and maybe even the caecum. The canine caecum harbours the highest amounts of SCFA's compared to other gut compartiments [83] and although rather small of size [84,85], the caecum demonstrates some motoric activity. It generates giant migrating complexes (GMC) which may serve the expulsion of caecal content into the colon [86]. When studying faecal descriptions of carnivores that do not possess a caecum, findings are contradictory. The ferret (*Mustela putorius*) does not have a caecum [87,88] and typically only produces hard stools when fed whole prey [87]. However, the panda (*Ailuropoda melanoleuca*) has no caecum and when fed a diet based on bamboo, sugar cane and gruel, they produce normal and mucous stools at various intervals [89], which seems to contradict the hypothesis that a caecum is a prerequisite for a separation mechanism in the hindgut, and therefore requires further study.

Be it stomach or hindgut that separates digesta fractions, if a separation mechanism would be apparent in dogs, we would expect the digesta fractions, i.e. soft and firm faeces, to transit differently through the intestinal tract based on other herbivore and avian species [3–5]. However, no pattern of marker excretion differences between firm and soft faeces could be observed in this study except for one dog (Fig 3a), indicating that a monophasic digesta movement may not always be the case. However, since this only occurred for one dog in one test period, this might be a coincidental observation.

The faecal dichotomy could occur due to reasons related to the dogs' behaviour or diurnal activity pattern. Dogs might have retained their faeces in the colon/rectum hence enabling more fermentation [56] or more water and electrolyte absorption [50]. However, one would not expect the faecal discrepancy to occur in an almost alternating pattern. Similarly, relating the faecal dichotomy to the activity pattern of dogs, i.e. diurnal rhythm, would not explain the alternating pattern that occured independently of the time of day.

As last, it could be that an ileal brake mechanism occurred [90], given the analogies of plant and animal fibre, and that the ileal brake kept back the larger material but not the fluids, leading to the faecal dichotomy. It could be that mucus produced in the hindgut is somehow related to the faecal consistency or fermentation difference. It is known that SCFA stimulate mucus release in the hindgut [91,92], possibly leading to softer stools. However, in order to come to a faecal consistency dichotomy (soft vs firm), SCFA production should initially have differed, hence suggesting fractionation of fermentable substances.

### Biological relevance

The question *how* the occurrence of a faecal consistency dichotomy comes about requires further investigation. As for the *why*, reasons remain highly speculative. As mentioned before, ridding the gut of the coarse indigestible compounds present in whole prey might enable carnivores to, apart from enzymatic digestion in the upper gut, efficiently use whole prey by enhancing fermentation in the hindgut (assuming the caecal hypothesis holds to be true). However, protein fermentation is also associated with the production of putrefactive compounds such as ammonia (NH3), phenols, indoles, aliphatic amines and sulphur-rich compounds [93], and the presence of indigestible compounds (i.e. hairs and bones) in the hindgut might serve as a bulking agent, forming a physical barrier between substrates and bacteria and filling the large intestine, tempering protein fermentation [17]. Hence, answering the *why* seems too early at this stage, but the possibility that digesta separation may simply be a consequence of normal colon peristalsis or gastric retention on structured diets, without any apparent function, should not be forgotten. Without making any precarious statements on biological relevance, feeding raw meat diets to domestic carnivores (e.g. BARF feeding (Bones and Raw Food)) is an increasing practice in domestic carnivore and raw meat diets are often associated with diarrhea [69], which might not be as straightforward as thought before, since (alternating) liquid faeces might be a physiological response to a raw meat diet (if infectious diarrhea is excluded).

A further elaboration of passage studies in which a fluid and solid particle marker (powder and beads of different sizes) could offer more clarity in the passage of different fractions associated with whole prey through the gut. Whereas it is common practice in studies on the digestive physiology in herbivores to compare the movements of fluids and particles in the digestive tract [2,6], it is rarely done in carnivores. Most likely, this is due to the impression that little differences are to be expected between the digesta phases, and hence such tests may have little physiological relevance. The comparison of fluid and particle marker however can yield insights into retention mechanisms. Similar feeding trials with a stronger dietary contrast in terms of structure are imposing. Furthermore, a design which enables a fasting period before and after feeding should offer more clarity in the linkage of a dichotomy with diurnal rhythm. Further characterisation of soft faeces in terms of microbiome, protein content and animal fibre levels is warranted.

## Supporting information

S1 Data Faecal characteristics of 6 beagle dogs 6 fed a fine or coarse diet in a latin square cross-over design. (XLSX)

S1 Fig Marker excretion patterns of the TiO_2_ marker for all six beagle dogs for both dietary treatments. Black circles = firm faeces (faecal score 1 to 3.5); White circles = soft faeces (faecal score 4 to 5).
(PDF)
