# Supplementary material for: Are carnivore digestive separation mechanisms revealed on structure-rich diets?: Faecal inconsistency in dogs (Canis familiaris) fed day old chicks
Source: PLoS One. 2018 Feb 12;13(2):e0192741. doi: 10.1371/journal.pone.0192741 (PMC5809083; doi:10.1371/journal.pone.0192741)

Dog 1 - Coarse diet

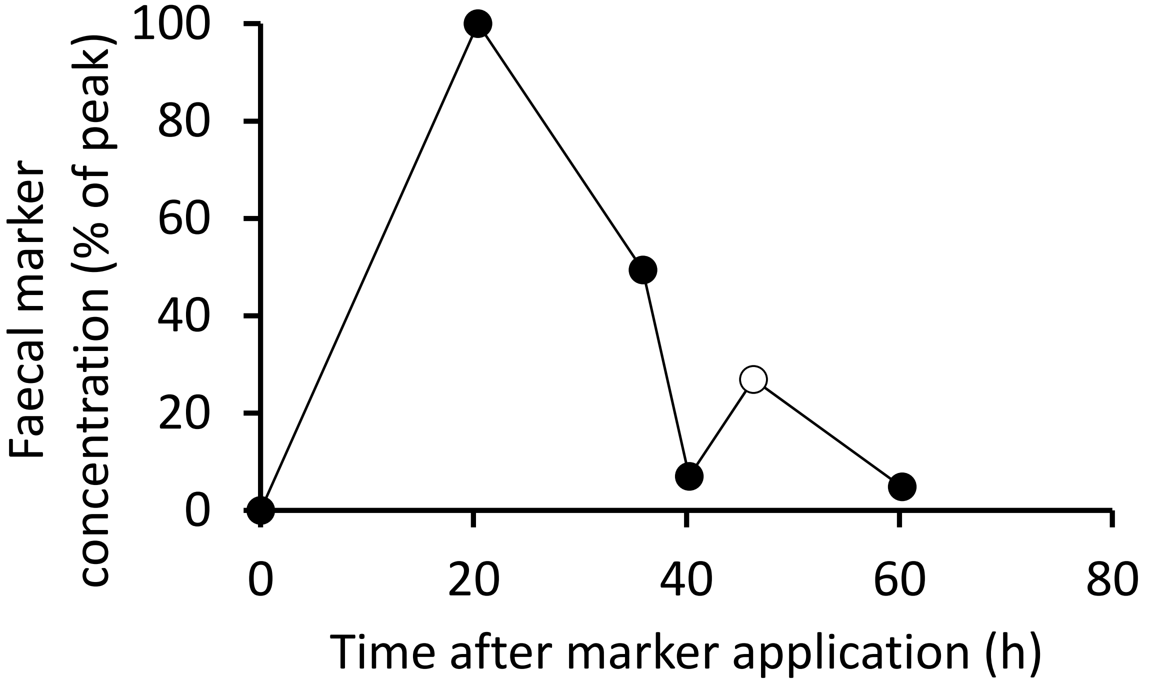

Dog 1 - Fine diet

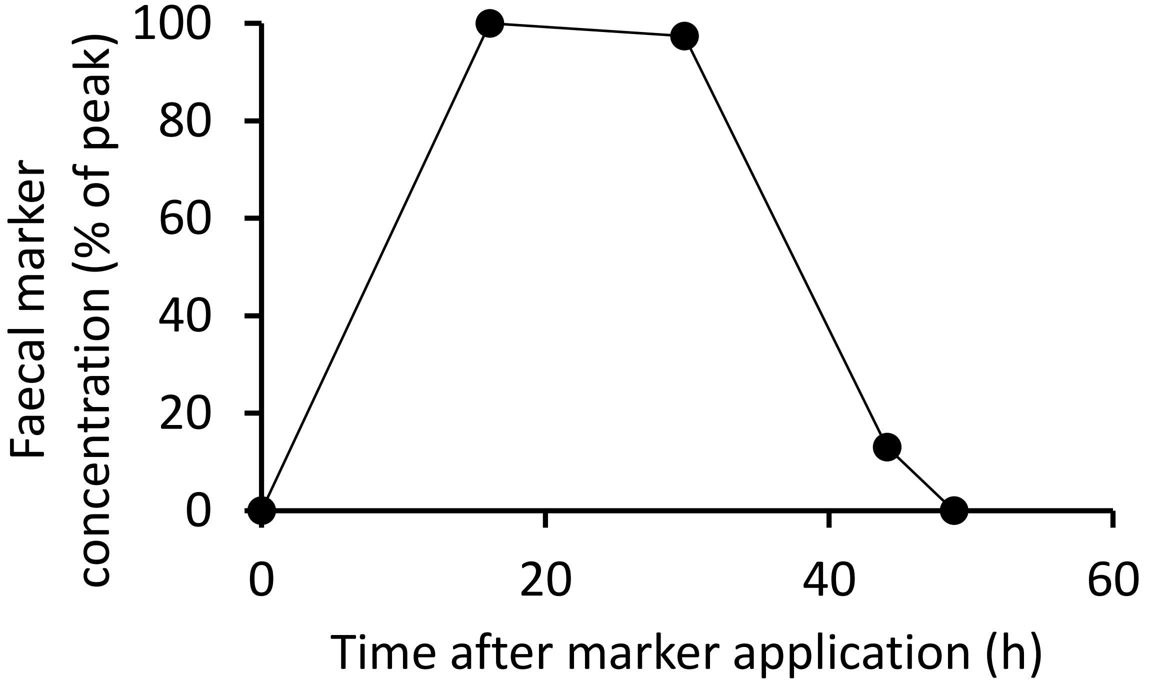

Dog 2 - Coarse diet

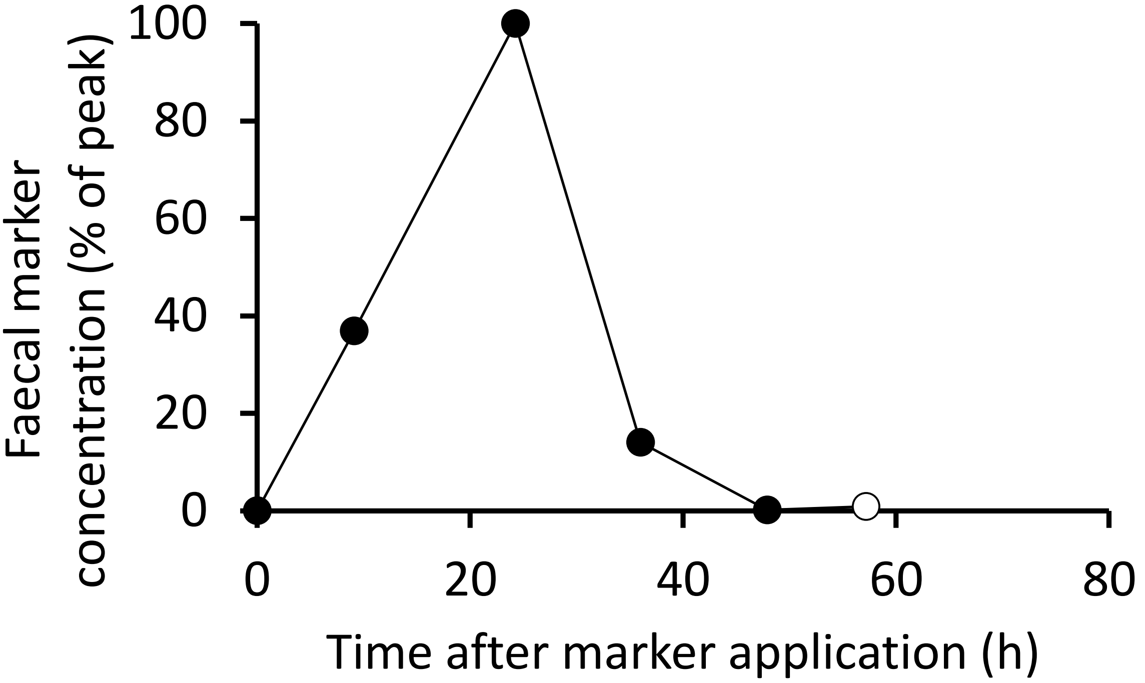

Dog 2 - Fine diet

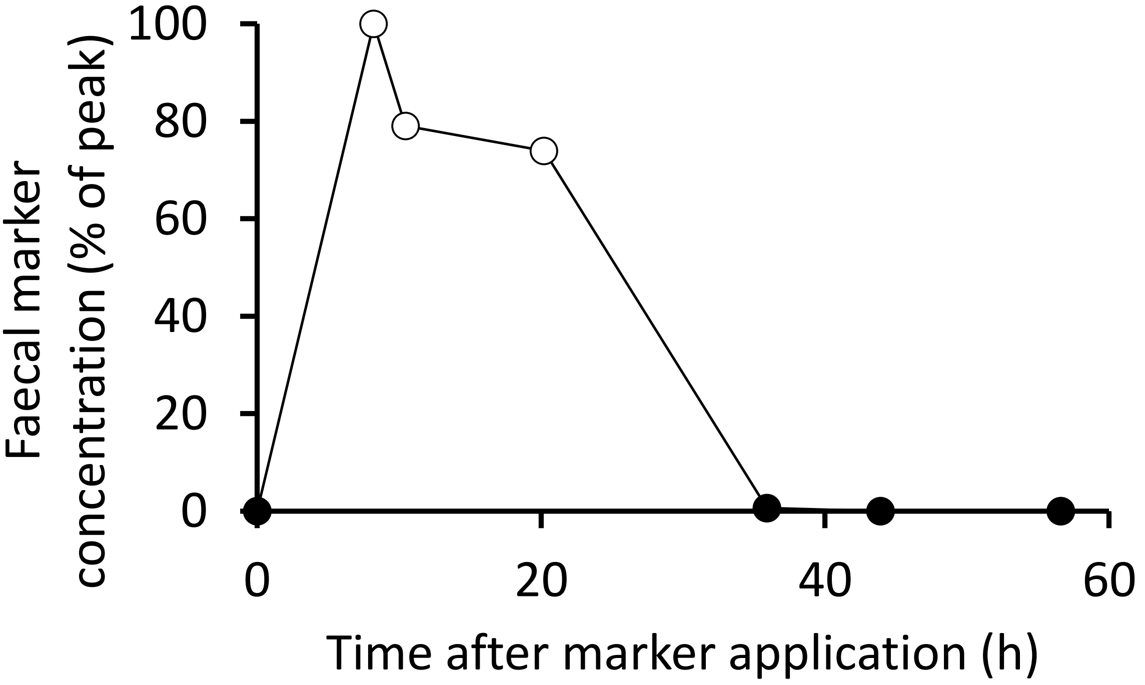

Dog 3 - Coarse diet

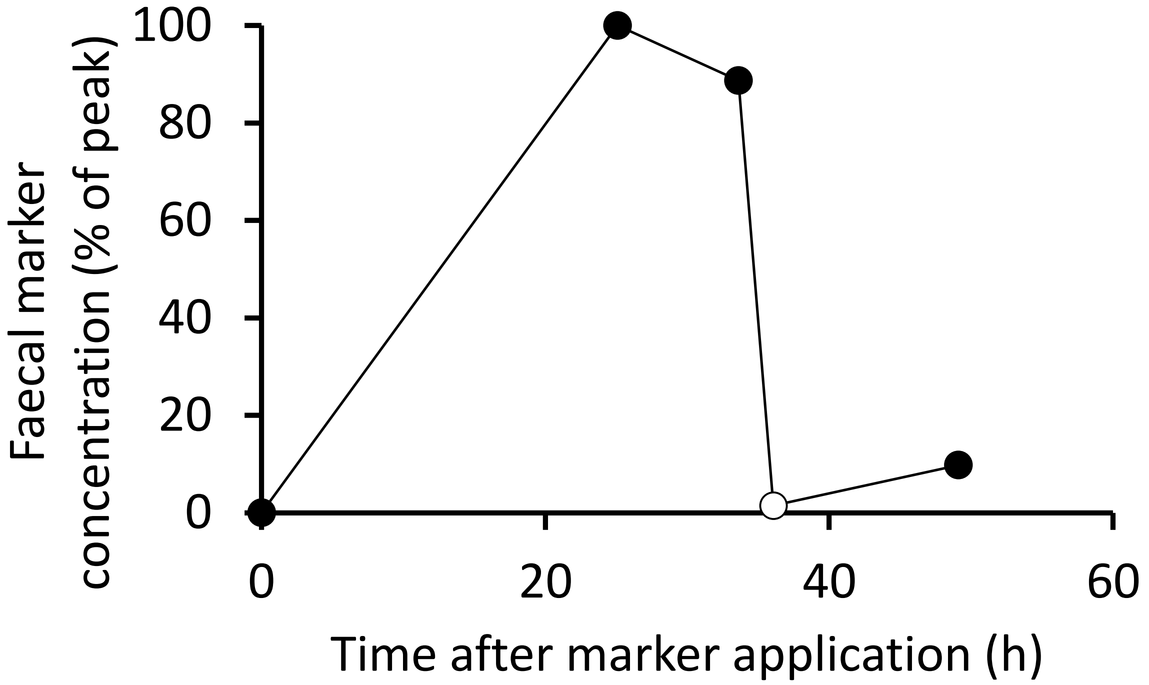

Dog 3 - Fine diet

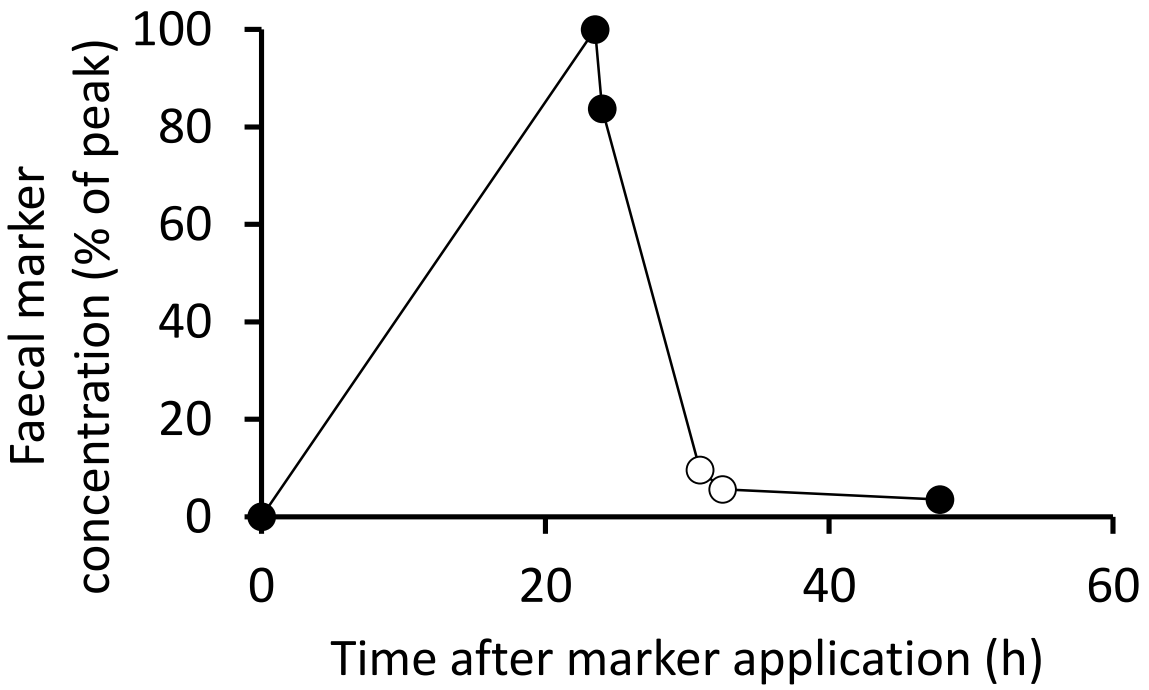

Dog 4 - Coarse diet

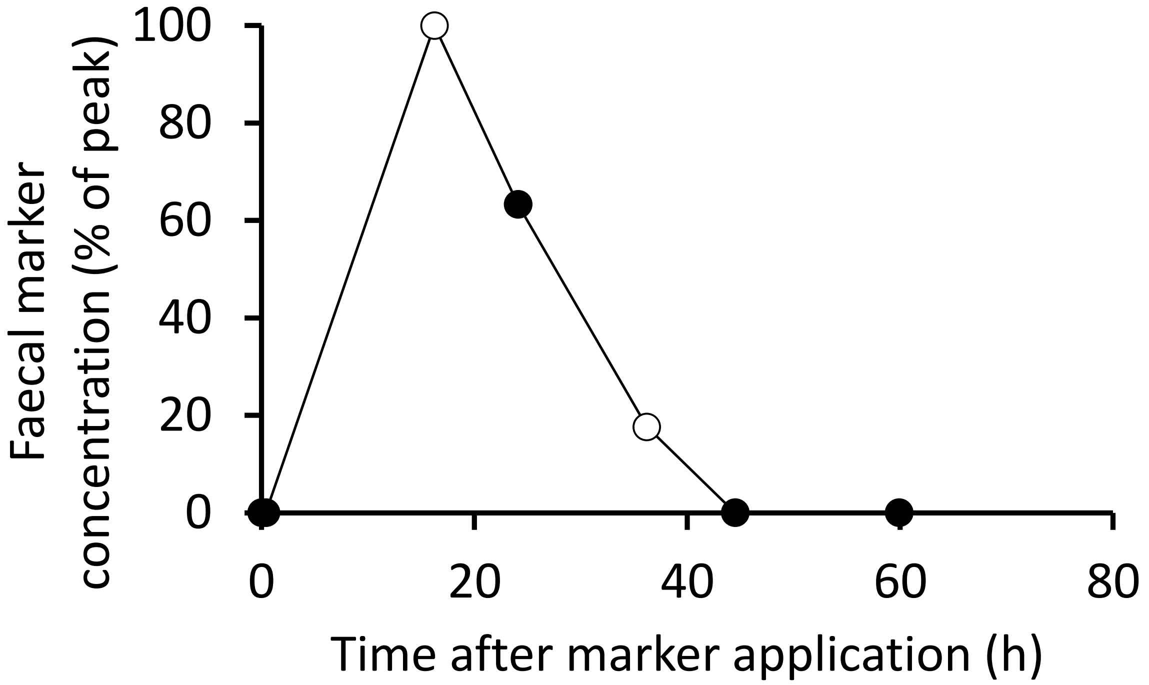

Dog 4 - Fine diet

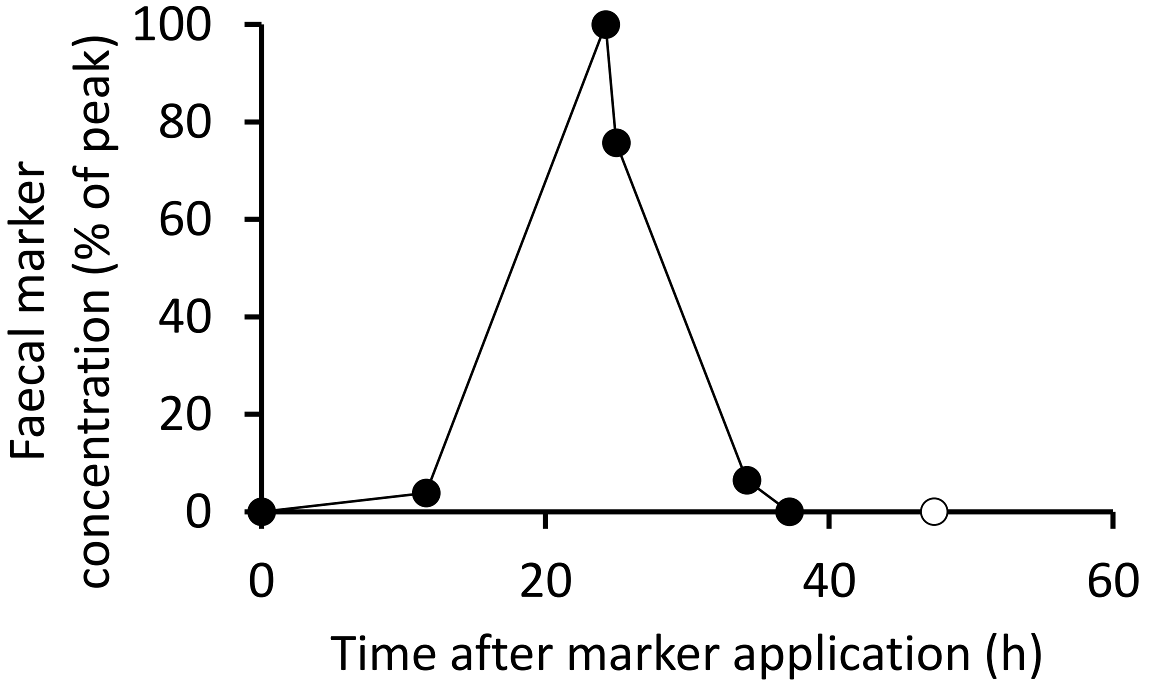

Dog 5 - Coarse diet

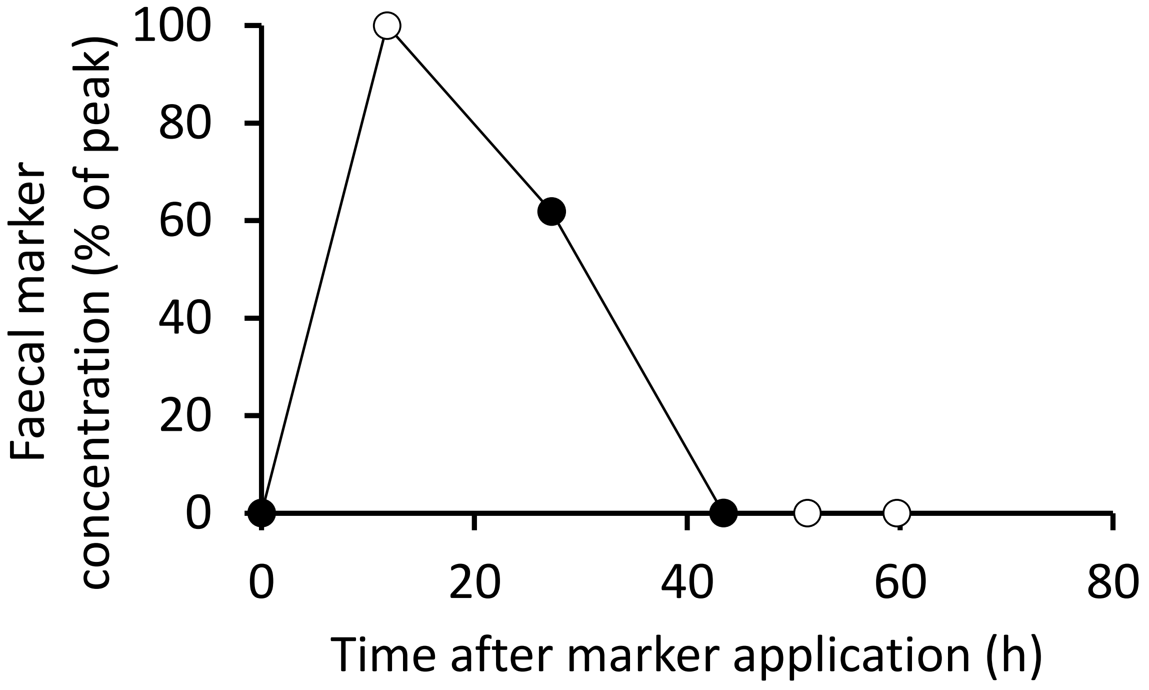

Dog 5 - Fine diet

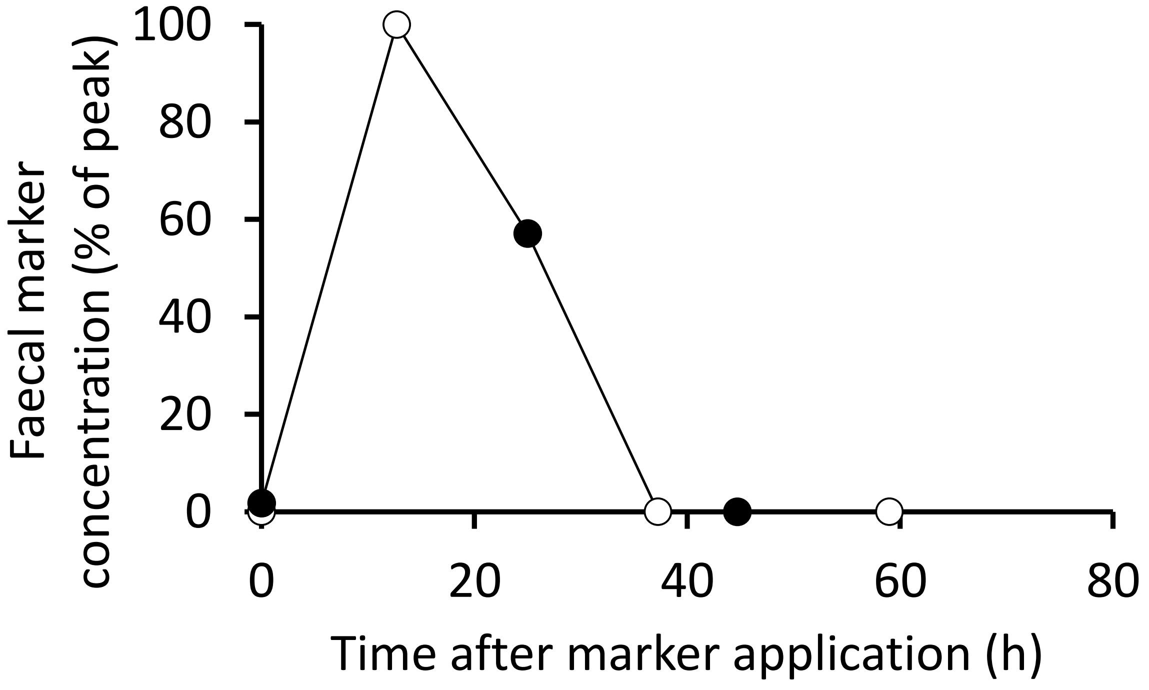

Dog 6 - Coarse diet

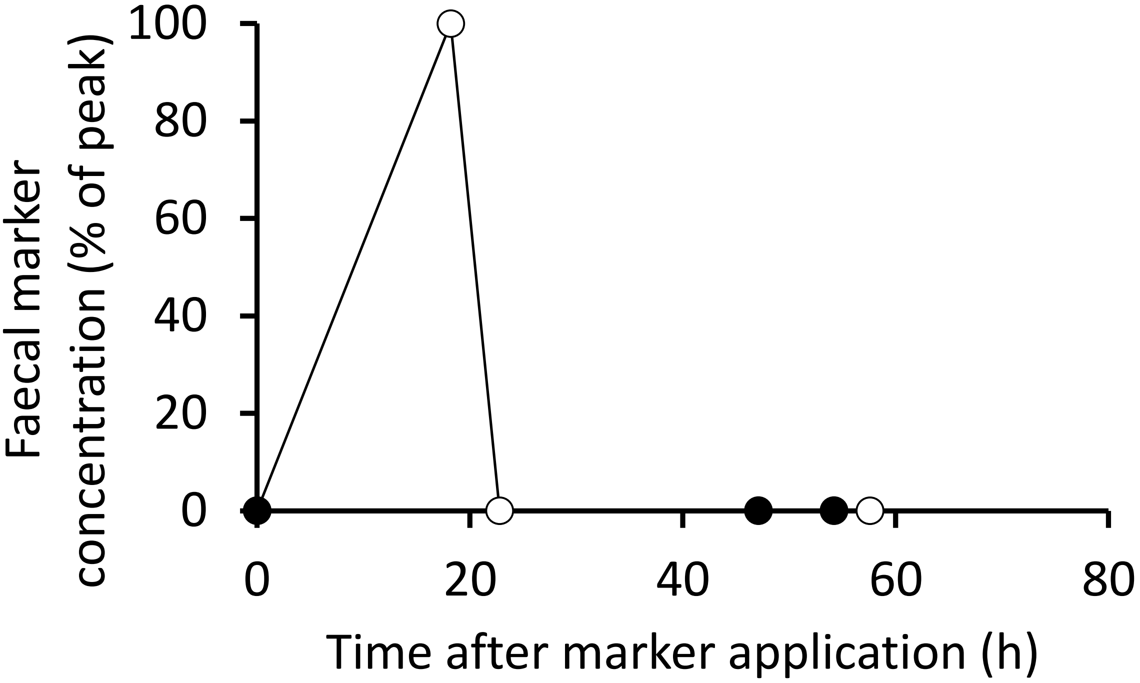

Dog 6 - Fine diet

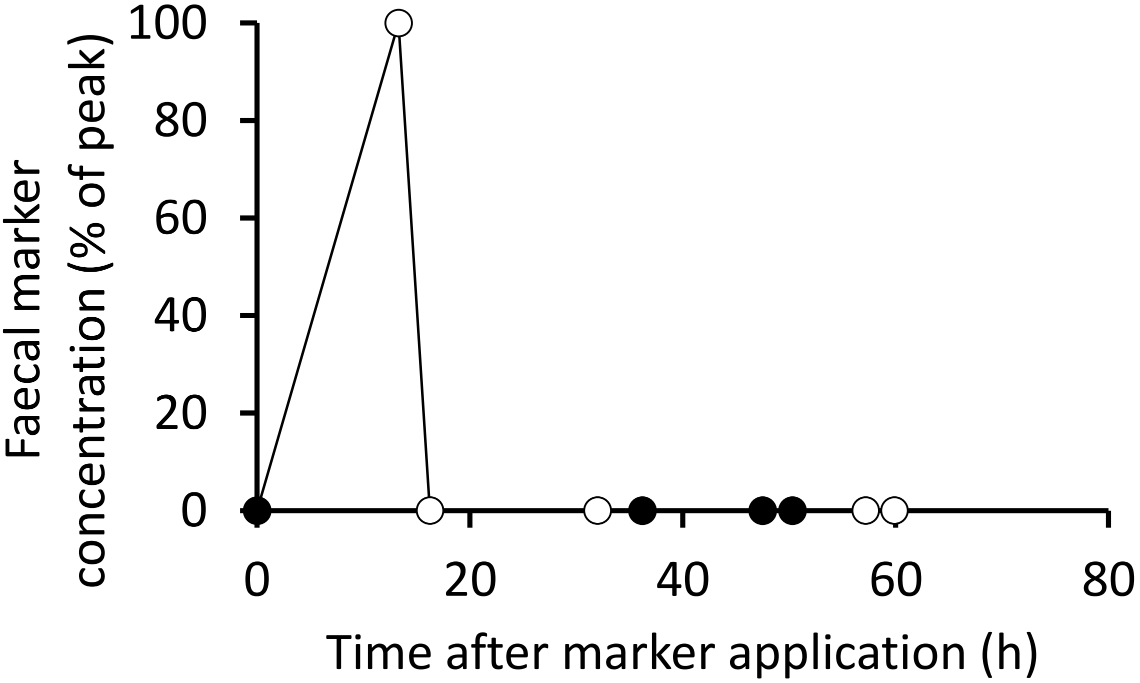

Supplement: S1 Fig — Black circles = firm faeces (faecal score 1 to 3.5); White circles = soft faeces (faecal score 4 to 5). (PDF) [file pone.0192741.s002.pdf]
